# Extended Reality in Neurosurgical Education: A Systematic Review

**DOI:** 10.3390/s22166067

**Published:** 2022-08-14

**Authors:** Alessandro Iop, Victor Gabriel El-Hajj, Maria Gharios, Andrea de Giorgio, Fabio Marco Monetti, Erik Edström, Adrian Elmi-Terander, Mario Romero

**Affiliations:** 1 Department of Neurosurgery, Karolinska University Hospital, 141 86 Stockholm, Sweden; 2 Department of Clinical Neuroscience, Karolinska Institutet, 171 77 Stockholm, Sweden; 3 KTH Royal Institute of Technology, 114 28 Stockholm, Sweden; 4 SnT—Interdisciplinary Center for Security, Reliability and Trust, University of Luxembourg, 4365 Esch-sur-Alzette, Luxembourg

**Keywords:** extended reality, neurosurgery, education, virtual reality, augmented reality, mixed reality, procedural knowledge, simulation, residents

## Abstract

Surgical simulation practices have witnessed a rapid expansion as an invaluable approach to resident training in recent years. One emerging way of implementing simulation is the adoption of extended reality (XR) technologies, which enable trainees to hone their skills by allowing interaction with virtual 3D objects placed in either real-world imagery or virtual environments. The goal of the present systematic review is to survey and broach the topic of XR in neurosurgery, with a focus on education. Five databases were investigated, leading to the inclusion of 31 studies after a thorough reviewing process. Focusing on user performance (UP) and user experience (UX), the body of evidence provided by these 31 studies showed that this technology has, in fact, the potential of enhancing neurosurgical education through the use of a wide array of both objective and subjective metrics. Recent research on the topic has so far produced solid results, particularly showing improvements in young residents, compared to other groups and over time. In conclusion, this review not only aids to a better understanding of the use of XR in neurosurgical education, but also highlights the areas where further research is entailed while also providing valuable insight into future applications.

## 1. Introduction

### Rationale

Neurosurgical residency training encompasses the acquisition of several years worth of both nominal and procedural knowledge as well as practical skills. Perfecting these skills requires continuous learning and hands-on training even beyond residency. However, the complexity of the procedures and the delicate character of the areas being operated on greatly limit young residents’ opportunities to train, which in turn further stretches their learning curve. Similarly to the field of aviation [1,2], simulation has provided neurosurgery with a “trial-and-error”-based method of learning without putting patients at risk.

**Figure 1:**
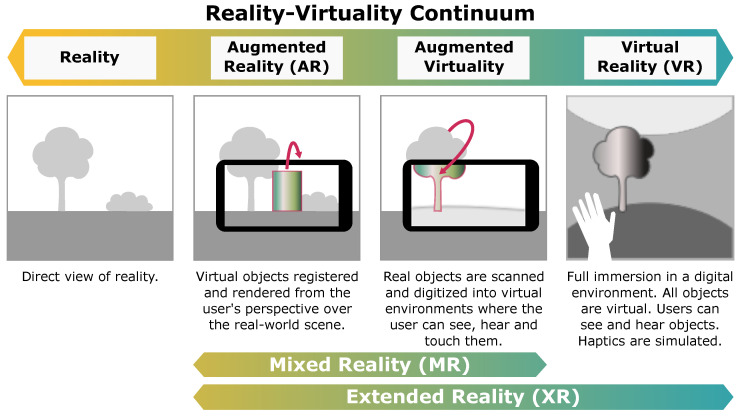
Reality–virtuality continuum. Spectrum of physical and extended reality, with virtual reality at one end and the real world at the other.

Neurosurgical simulation can be performed on cadaveric [3], animal, or physical 3D models [4], or through the adoption of extended reality techniques [5,6]. Of these, only the latter has the potential to cost-effectively offer seamless and unlimited use, together with a realistic reproduction of physical reality. Extended reality (XR) refers to the spectrum of applications that fuse (i.e., superimpose) virtual and real imagery, for instance, 3D models with live camera feeds, as shown in Figure 1. At one end of the spectrum lies the physical reality and at the other end lies virtual reality (VR), where all visual imagery is computer-generated and the user is fully immersed in a digital environment. Between the extremes of the spectrum we can also find mixed reality (MR, not to be confused with the medical acronym widely known for magnetic resonance), corresponding to physical reality with the addition of virtual objects. A popular subset of MR technologies is augmented reality (AR).

Within the medical field, XR has been applied in a variety of contexts other than educational, for example, in preoperative surgical planning [7,8], in intraoperative navigation [9,10,11], for improving patient care through rehabilitation [12,13], in patient education [14,15], and informed consent [16]. Research employing XR technologies has also focused on surgical performance in critical conditions, for instance, in cases of sleep deprivation [17].

Regarding neurosurgical education, it is now evident that practicing in a virtual environment adds valuable aspects compared to a classical observation-based way of acquiring procedural knowledge. Training in an XR setting may also provide exposure to a nuanced library of cases to better prepare the student for the diversity of real cases [18,19]. In addition, these techniques allow for remote education, where learners and teachers are located in different places and the communication between them (i.e., the transmission of knowledge) takes place at different times. Consequently, a higher number of participants can potentially virtually attend neurosurgical operations from all over the world, while in the comfort of their own homes and without having to “compete” for a spot in the operating room (OR), a scenario explored in previous research using 360° cameras [20]. Implementing such an asynchronous and distributed educational tool, by recording surgical procedures and enabling viewers to control their video playback, can serve to promote education, especially in areas of the world where access to relevant expertise is limited [21,22].

Neurosurgery partly owes its complexity to the intricacy of the anatomy involved, hence the need for a deep neuroanatomical knowledge. Studies have shown that learning anatomy while immersed in a virtual environment might aid in retention and recall of topographic [23] as well as operative anatomy [24,25,26]. Virtual volume rendering technologies are sometimes used to supplement XR in conjunction with accurate 3D models, or haptic feedback devices. The aim is to realistically simulate surgical procedures through illusions of tissue deformation. By displaying its behavior when sufficient force is exerted on it using a (virtual or physical) tool, or by providing users with tactile feedback, a more holistic experience is created. When haptic devices are used in an XR application, a whole new aspect of performance assessment is revealed, mainly through tracking of force, motion, tremor, and hand ergonomics [27,28,29]. Assessment of performance and dexterity with haptic devices in users has been employed to select future neurosurgical residents [30,31], and to determine skills and track progress in current residents’ training [32].

More in general, we define user performance (UP) as a set of metrics that quantifies the efficiency and effectiveness of a surgical simulation carried out on an XR system. It includes, but is not limited to, measures of accuracy and precision, as well as outcome of the surgery, speed in the execution, and number of attempts per user. Alongside UP, user experience (UX) is an essential aspect of evaluating an XR application or simulation device, as it accounts for the way test subjects perceive the events, interactions, and feedback associated with the simulation environment and tools. We distinguish several categories of UX metrics in order to be able to formally group indicators across different studies when applicable: usefulness, the user’s opinions on the impact and effectiveness of the proposed application; self-assessment, the appreciation and evaluation of the user’s own performance; haptic interaction, related to the system’s tactile input and output; ease of use; system feedback, the environmental response to user interaction; comfort—i.e., ergonomics; time requirements (to perform actions and receive feedback); engagement; immersiveness, the “sense of being there” in the virtual environment; and realism, the visual (and tactile, when applicable) fidelity of the application with regards to real surgical procedures [33,34].

The adoption of XR technologies in neurosurgical education promises to change the pedagogical landscape, improving the quality of teaching and the efficacy of procedural knowledge acquisition. The rapid increase in the number of studies performed and published on this topic is clear evidence of such a trend in modern medicine, and particularly in neurosurgery [35]. An arising interest has been shown in novel technologies that allow residents to participate in complex neurosurgical procedures and tasks from early years of training. In fact, the results of a recent survey distributed to neurosurgery program directors to collect their opinions on simulation as an educational tool in the USA concluded that simulation is considered to be essential for practice alongside the more conventional training methods [36].

The present systematic review is intended to study and discuss the recent research landscape on the use of XR technologies in neurosurgical education, with a focus on cranial neurosurgical practices. In this context, we aim at adding to the current understanding of the field and provide useful insights for both clinicians and residency program directors who are interested in what XR technologies have to offer. In particular, our goal is to answer the following research questions:

1. What kinds of cranial surgical procedures is recent research focused on?
2. Is research on the topic localized in specific geographical areas of the world, or is it evenly distributed?
3. Are users benefiting from the use of XR technologies in education, and appreciating their fidelity to real-life scenarios? In other words, are proposed applications useful and realistic according to the users?
4. What metrics are used to assess the impact of these technologies on performance, usability, and learning curves of test subjects? Are such metrics employed across multiple studies, or are they related to a specific experimental setup?
5. What devices are used in recent research on the topic?

Ultimately, through the present review we seek to both detect trends and uncover knowledge gaps within this research area, in order to support future endeavors in the field. Findings presented here may highlight opportunities for related work and raise important questions for future studies.

## 2. Methods

This systematic review is reported in accordance with the Preferred Reporting Items for Systematic Reviews and Meta-Analyses (PRISMA) guidelines [37]; the related 2020-PRISMA checklist is provided as Supplementary Material (Supplementary File S1). The review protocol was registered within the International Prospective Register of Systematic Reviews (PROSPERO) (Registration ID: 319508 and date of registration: 20 March 2022). The record was constantly updated in the case of any change to the design of the work.

### 2.1. Eligibility Criteria

All empirical studies published between 2013 and 2022, written in English, and focusing on education through XR technologies in cranial neurosurgery were eligible for inclusion in the present systematic review (Table 1).

### 2.2. Types of Studies

The systematic review includes original, experimental, peer-reviewed studies, regardless of publication status. Non-empirical studies presenting XR techniques (e.g., pipelines, systems, know-how) without any user studies for validation purposes are excluded.

### 2.3. Types of Population

No restrictions to inclusion were made based on the training level or experience of participants. This means that test subjects recruited in the included user studies can be medical students (MSs), residents, or experienced neurosurgeons.

### 2.4. Type of Intervention

Only articles focusing on the use of extended reality applications for cranial neurosurgical education were considered for inclusion in this review. Studies addressing the use of XR within other contexts such as patient education, informed consent, preoperative planning, or intraoperative navigation were systematically excluded. The focus of this review relied on commercial, off-the-shelf stereoscopic displays; hence, all studies introducing or employing custom devices that are not available on the market were excluded.

### 2.5. Types of Comparators

There were no restrictions with respect to the type of comparator. This means that control cases can include between-subject conditions, which compare members of the same population, as well as within-subject conditions, which quantify changes in UP/UX for each test subject, and longitudinal studies, which focus more on the long-term impact of the proposed application.

### 2.6. Types of Outcome Measures

The main outcomes of interest were measures of performance and usability assessed objectively and subjectively by users interacting with different systems.

### 2.7. Databases and Search Strategy

Five electronic databases covering medicine and technology were used: PubMed, Scopus and Web of Science for medicine; IEEE Xplore and ACM Digital Library for engineering and technology were searched. To ensure reproducibility of our findings, an extensive and elaborate description of the steps that went into the creation of our search strategy (Supplementary File S2) as well as a detailed description of the query (or queries) applied in each engine is provided (Supplementary File S3). The search strategy applied to the present review in February 2022 limits results to the last decade (2013–2022), and only includes papers written in English.

### 2.8. Study Selection

**Figure 2:**
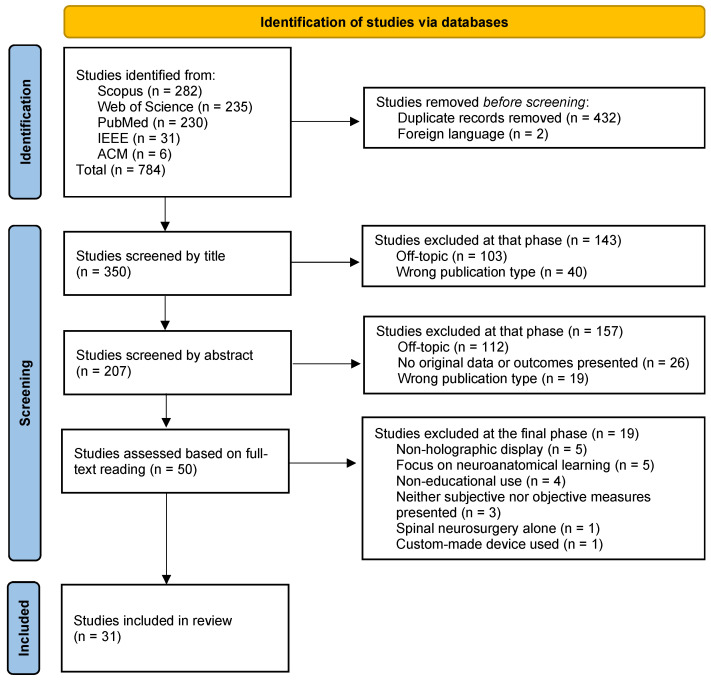
Study selection (PRISMA) flow chart. Summary of the selection process applied in the present review, divided into identification, screening, and inclusion phases.

The search yielded a total of 784 papers across databases. Once retrieved, the records were uploaded onto Rayyan [38], where manual deduplication was performed, leaving 352 articles. It was then noted that two articles were written in foreign languages without translation, which resulted in their exclusion. The remaining 350 records were then screened by two independent and blinded reviewers (V.G.E. and A.I.), first by title and subsequently by abstract. In the final step, full texts of the 50 remaining articles were extracted and separated into three groups of 16 or 17 articles, so as to assign two of them to each of three blinded and independent reviewers (A.I., V.G.E., and M.G.). This way, each group of articles would be reviewed by two different reviewers. After each step, conflicts were solved through both discussion between the involved parties and consultation of a fourth reviewer (M.R.). The entire process yielded a total of 31 studies deemed eligible for definitive inclusion in this review, as illustrated in the PRISMA flow chart shown in Figure 2.

### 2.9. Data Extraction

Data from selected records were extracted using a predefined template including (1) general information, i.e., title, first author, journal, publication year, location, etc.; (2) population characteristics; (3) intervention characteristics, i.e., applicability and end-use of the technology, specific domain of application, kind of device used, use of haptic devices, combination of other models; (4) study characteristics and comparators, study design (cross-sectional, case control, or cohort studies), controls; (5) outcomes, i.e., subjective measures, surveys, objective performance metrics; and (6) results and conclusions, i.e., positive or negative results and short summary of the final study outcome.

### 2.10. Data Synthesis and Risk of Bias Assessment

Although all included studies revolve around the use of XR in cranial neurosurgical education, the procedures simulated in these articles are diverse, from tumor resections to ventriculostomies and trigeminal rhizotomies. This variability in domain of application together with the heterogeneity among both the devices used and the study designs, hinders the performance of a meta-analysis. Consequently, we chose to adhere to a narrative synthesis of the data to describe the body of evidence gathered around the topic of interest, report trends, and highlight the gaps within the literature. Since this systematic review focuses on the use of XR in neurosurgical education specifically, we chose the Newcastle–Ottawa Scale-Education (NOS-E) [39] as a risk of bias assessment tool tailored to assess medical research quality. According to it, a score on a scale from 0–6 was allocated to each study based on a specific set of items.

## 3. Related Works

A quick search of both PubMed and PROSPERO revealed seven systematic reviews revolving around topics close to the one addressed by our review [5,40,41,42,43,44,45]. Two of them [40,41] address the use of simulation models as a tool in neurosurgical education, including all kinds of simulation systems such as 3D-printed, cadaveric, and animal models. However, both reviews collected a limited number of studies that specifically targeted XR-based techniques. Dadario et al. [42], on the other hand, reviewed the literature addressing the uses of XR in neurosurgery, while specifically focusing on patient outcomes, a topic of interest which is briefly addressed in our review. Chan et al. [5] performed a systematic literature review by uniquely retrieving articles which address the use of XR tools in assessment of neurosurgical trainee performance, an aspect that is of high relevance to the current era but only partially covers the aspects which we intend to cover in this review. Our aim of reviewing the literature surrounding the specific topics of cranial neurosurgical education, as well as XR-based simulation techniques, is shared with Mazur et al. [43]. However, in their review, only a modest number of studies (n = 9) were included, even encompassing both educational and preoperative applications of VR techniques. Although the 2016 review by Barsom et al. [44] and the 2022 review by Innocente et al. [45] studied the application of holographic techniques within neurosurgical education among other medical fields, their overall scope was strictly limited to augmented reality techniques while neglecting all other technologies belonging to the XR spectrum. In summary, to the best of our knowledge, this systematic review is the first to specifically target the educational application of XR techniques within the field of cranial neurosurgery, while still collecting the highest number of studies, compared to the aforementioned studies.

## 4. Results

### 4.1. Characteristics of the Included Studies

Of the 784 studies identified, only 31 were finally included in this systematic review (Table 2), with a mean NOS-E score of 3.23 ± 1.17 (±SD) (Supplementary File S4). Thirteen of them originate from Canada, 10 from the USA, and one from Mexico, which makes a total of 24 in North America alone (77% of all papers). The remaining seven studies were carried out in the UK, Italy (n = 2), China (n = 2), South Korea, and France.

In the absolute majority of the included studies, participants belonged to the medical community and were either medical students, residents, or expert neurosurgeons. Only two articles (6%) relied solely on participants without any medical training. The studies, however, significantly diverged in the neurosurgical procedures simulated, with the two most common being tumor resections (n = 14, 51%) and ventriculostomy placements (n = 6, 19%). Most resection simulations were of unspecified tumor type, while only a few studies presented tumor-specific simulations, including meningioma and GBM resections. Aneurysm clipping, a common procedure in neurosurgery, was only simulated in three studies (10%). Other procedures, such as tumor localization and access, trigeminal rhizotomy, endoscopic surgery, cauterization, or bipolar hemostasis, were each attempted once, while the rest of the studies involved unspecified surgical tasks that can be applied to several different kinds of procedures.

Among the included studies, the use of NeuroVR (National Research Council, Montreal, QC, Canada)—previously known as NeuroTouch—was most common. In fact, 16 studies (52%) employed this device when studying VR technologies in the field of neurosurgery. Another frequently used technology was ImmersiveTouch (ImmersiveTouch, Chicago, IL, USA), which was the case for seven (23%) studies, whose stereoscopic 3D display is similar to that of NeuroVR. Seven of the remaining studies employed other stereoscopic devices, including the HoloLens (Microsoft, Redmond, WA, USA), the Oculus Quest 2 (Meta, Menlo Park, CA, USA), and the HTC VIVE Pro (HTC Corporation, New Taipei City, Taiwan) (Table 2). In one study the device employed was not specified. Of the devices employed, 23 were based on stationary and flat monitors (74%), while eight used HMDs (26%). Additionally, it was found that the majority of studies relied on VR technologies (84%), while only a minority utilized AR (10%), and even AV ones (6%). Twenty-six articles (84%) also presented the use of haptic feedback technologies combined with the visual holographic simulation used.

Broadly, the studies can be categorized to focus on (1) learning, acquisition of new skills; (2) practicing, to maintain or improve existing skills; (3) the assessment of skills. The use of XR as a tool for learning was investigated in nine studies (29%), while 10 (32%) looked at utilizing XR for practicing surgical skills, and 12 studies (39%) focused more on its use for the assessment of participant skills and surgical dexterity. To determine the benefits of XR in the field of cranial neurosurgical education, studies mainly focused on two types of outcomes: user performance (UP) and/or user experience (UX). UP denotes objective metrics of simulation-related dexterity and hand–eye coordination skills, while UX denotes subjective metrics of usability and appreciation of the interfaces. UP was evaluated in 18 (58%), UX in eight (26%), and both UP and UX in five (16%) of the studies, respectively.

### 4.2. User Performance (UP)

We found that the type of UP metrics used varied depending on the procedure studied. In all studies simulating tumor resections, the metrics were automatically computed by the software [27,28,31,47,50,53,54,57,58,59,70,71]. These metrics can be grouped under three tiers, where tier 1 metrics included the percentage of tumor resected and the volume of healthy tissue removal, tier 2 metrics included the time required to complete the task and the path length of different instruments, and tier 3 covered kinematic metrics such as measurements of force applied. Even though not every study adhered to this specific classification system, nearly all of them included most of the named metrics. A few studies [31,47,54,58,70] even incorporated advanced derived metrics such as tumor resection effectiveness—calculated by dividing the volume of tumor removed by the volume of healthy tissue removed—or the tumor resection efficiency—calculated by dividing the volume of tumor removed by the path length for each hand. Three studies (12%) [47,58,70] also looked at the volume of blood lost during the tumor resection simulation. Among these studies employing advanced derived metrics, there were none that used subjective metrics to assess the performances of participants.

Five articles studied UP in ventriculostomy placement. Four of these (80%) used objective metrics specific to the simulated procedure, including length of the procedure, accuracy measures, e.g., distance from the catheter tip to the target and depth of the catheter, and success measures, e.g., number of first-attempt successes [19,51,60,65]. These metrics were typically computed automatically by the software utilized, except for one study where experts were assigned the task of estimating the UP to create an individual score [60]. On the other hand, a single study focusing on UP in ventriculostomy placement adopted a more subjective approach instead of using objective performance metrics, by allowing participants to fill in a survey assessing memory retention with procedure-specific questions [64].

The UP metrics studied in the two aneurysm clipping simulation studies [62,68] included measures of position and orientation of the clip. However, while one of the studies focused on kinematic performances with assessment of forces used, path length, velocity, and acceleration, as well as jerk and contact frequency [68], the other concentrated on outcome measures, such as presence of residual aneurysm, patency of distal branches, and clip choice appropriateness, in order to estimate an individual score based on the clip evaluation scale [62]. The outcome-centered approach of the latter likely was related with the design of the study which also later involved performance assessment of live-patient procedures, and comparison between the residents with XR simulator training vs. those without. Indeed, the tracking of clinical outcome measures even during simulation would give a better estimation of the resident’s performance during live-patient surgery than kinematic metrics. During the live-patient procedure, the residents were further assessed based on length of surgery, length of hospitalization, aneurysm occlusion, patency of the normal vessel, and intraoperative complications [62]. In the remaining studies reported here (n = 6), UP was assessed for a diverse range of different procedures, such as trigeminal rhizotomy [66], localization of tumors [55] or objects [61] in the brain (without resection), endoscopic surgery [69], microsurgical tasks [49], and cauterization [30].

Eight of 26 studies (31%) adopted an “XR vs. non-XR” controlled design, assessing and comparing UP between groups assigned to different conditions. The use of XR (VR in six cases and AR in two) was associated with improved UP and contributed to better outcomes. Two studies adopting the “XR vs. non-XR” design [62,69] and one of the longitudinal studies [19] tested, and successfully validated, the hypothesis that XR training of residents could improve outcomes on live-patient subjects when compared to residents that were not trained with XR. Additionally, there were five studies—two on ventriculostomy placement, two on tumor resection, and one on endoscopic surgery [19,58,59,65,69]—that adopted a longitudinal design to address the participants’ individual improvement based on UP metrics.

One way of testing the realism of different XR interfaces is by looking at the relationship between overall UP and training level. Arguably, these variables are related, with better performances associated with the more experienced users. In the virtual world, systems that are able to reproduce similar changes in overall UP based on training level would typically have features that are closer to reality. We found 16 studies that investigated performance differences based on level of training or experience (Table 3). Differences in UP favoring the more experienced participants were found in all studies except one [67]. As expected, these UP differences were not consistent across all metrics assessed [57,58,59,65], and there were outliers among the less experienced who clearly outperformed their peers, and at times, even their superiors [31,57].

Finally, a small number of UP studies (n = 4) [19,58,59,65] assessed improvements in participants during a series of sessions (longitudinal design) while also looking at differences across training level or experience, mainly to test the hypothesis that less experienced users benefit more from the technology. This hypothesis was validated in two studies [58,65], while a single study showed mixed results [59]. In the last of the four studies, the absence of data on UP of experienced residents at follow-up did not allow for conclusions to be drawn, even though the authors reported that “residents felt that the simulator would be most helpful for novice residents” [19].

### 4.3. User Experience (UX)

All studies assessing UX employed surveys to collect test subjects’ subjective experience at different—and sometimes multiple—stages in the proposed user studies. These surveys can either be standardized, validated formats that allow for a quick comparison across multiple studies, or custom, tailor-suited sets of questions and associated metrics aimed at capturing specific details which might not be usually included in more generic questionnaires. Only one study (15%) [51] employed standardized questionnaires, namely, the System Usability Scale (SUS) [72] and the NASA Task Load Index (TLX) [73], to assess perceived usability and workload respectively. All other studies adopted custom questionnaires designed by the authors, often with the help of expert neurosurgeons. UX-related questionnaires can be categorized based on how they reflect usefulness (n = 9, 69%), self-assessment (n = 4, 31%), haptic interaction (n = 4, 31%), ease of use (n = 3, 23%), system feedback (n = 4, 31%), comfort (n = 1, 8%), time requirements (n = 1, 8%), engagement (n = 1, 8%), immersiveness (n = 2, 15%), and realism (n = 9, 69%).

The first and last categories—usefulness and realism—appear to be the most popular categories of UX questionnaire items (Table 4). In the nine studies reporting results on perceived usefulness of the proposed application [19,46,47,56,57,60,62,63,67], the vast majority of test subjects gave positive ratings to this specific aspect. Three studies (33%) [46,56,62] report differences between subject groups, with less experienced participants having overall more positive opinions towards the technology than more experienced ones. Study-specific questionnaires were used to assess realism. Five articles out of nine (56%) refer to the realism of the anatomy [46,52,63,67,68], while three (33%) distinguish between visual and sensory realism [19,47,57], one (11%) includes the realism of the surgical tools [52], three (33%) more broadly address the quality of the simulation [52,62,67], two (22%) focus on the haptic force feedback [67,68], and one (11%) refers to the realism of the view perspective [68]. The differences between subject groups are in this context less definitive compared to the rated usefulness of the applications: two studies reported a higher perceived realism in more experienced participants [62,68], while one reported a higher realism in less experienced ones [46] and one resulted in no significant differences between subjects [52].

Additional comparisons between groups were mentioned in four of the 13 total studies (31%): Alotaibi et al. [47] claimed that “junior residents self-evaluated their performance much higher than senior [residents]” in the context of tumor resection simulation; the second [52], that “prior experience with simulation and prior experience with assisting endoscopic third ventriculostomy (ETV) procedures did not have a significant effect on the participants’ perception of the simulators”; the third [56], that “residents also appeared to require less time to master the haptic device” when simulating bipolar homeostasis; the fourth [62], that “[residents] showed a higher level of appreciation for the customization of the surgical approach and corridor as compared with the expert surgeons”, when using the proposed aneurysm clipping application.

Eleven of the articles assessing UX did not employ standardized questionnaires, but proposed novel questionnaire items in order to investigate the quality of test subjects’ experience. Among these questionnaire items, it is not uncommon that part of them are related to specific aspects of the type of surgical operation that is being simulated. Including them can be useful in focusing research only on the relevant subject of interest, which is to assess the potential impact of the proposed application on users’ learning curve. The investigated surgery types were aneurysm clipping, tumor resection, ventriculostomy, and bipolar homeostasis. For aneurysm clipping (n = 3, 27%), items address preoperative preparation and planning [46,62], anatomical structure identification [62], consistency with real practices [62,68], and task difficulty [68]. For tumor resections (n = 4, 36%), items address task difficulty [47,57], metrics appropriateness [57], and consistency with real practices [67], while one study [53] reported questions that were not relevant to the surgical aspect. For ventriculostomy (n = 3, 24%), items address consistency with real practices [52], task difficulty [52], anatomical structure identification [19,60], and preoperative preparation and planning [19,60], as well as anatomical structure identification [60]. For bipolar homeostasis (n = 1, 9%), the only study [56] reported no surgery-specific questions.

In addition to the very popular Likert-scale items [74,75], which measure the participant’s agreement with statements along a bipolar scale and were employed by all 13 articles, a few of them employ other quantitative or qualitative metrics in the proposed surveys. In particular, two studies (15%) [46,56] included dichotomous (“yes or no”) items in their questionnaires, while four of them (31%) [19,52,57,63] included open questions to collect comments and suggestions. Two other studies (15%) [60,62] employed both kinds of items in their questionnaires. More specifically, dichotomous items address a variety of different aspects that are not always directly concerning UX, from the chosen action plan during an operation [60,62], to the previous experience of the participant [56], to visual feedback provided by the investigated application [56,60].

From the perspective of effectiveness and impact of the proposed applications, we found that three articles (23%) mentioned somewhat inconclusive results and pose that either further validation [56], additional expert input [54], or employing more traditional educational methods [60] could improve the usefulness of the proposed applications. On the other hand, the remaining 10 articles (77%) positively refer to the validity and efficacy of the related novel applications, focusing in particular on realism and usefulness as reported by test subjects. Additionally, we found that seven out of 13 studies (54%) explicitly address the potential impact of the proposed applications on didactic practices when reporting conclusions on UX metrics, and all of them do so with positive connotations. More specifically, one article (14%) reports positive outcomes for anatomical knowledge [52], three (43%) for training [19,56,60], three (43%) for skill acquisition [56,62,67], and two (29%) for educational assistance [60,63].

## 5. Discussion

This systematic review explored the use of XR in cranial neurosurgical education, in order to detect trends and uncover knowledge gaps within the research area. We found that the increasing volume of research on the application of XR in the field of neurosurgery does not necessarily coincide with equivalent geographical contributions. To the contrary, research on the topic seems to be mainly localized in North America (especially Canada and USA), where most of the devices employed in present studies are manufactured. Although off-the-shelf XR systems, and especially HMDs, may be accessible anywhere in the world, access to state-of-the-art advanced simulation technologies—which were represented in the majority (74%) of the studies included in this review—is limited in developing countries [76,77]. This is, in fact, concerning, as developing countries are oftentimes pointed out as an important beneficiary of XR technologies [21]. Nevertheless, we can compare the overall spread, market size, and pool of potential users between devices such as NeuroVR and ImmersiveTouch, which are built for the specific purpose of surgical simulation, and other commercial HMDs such as the HoloLens and HTC VIVE. It is clear that by adopting more easily available and widespread technologies in the implementation of XR educational applications, the digital divide as well as the economic barriers currently challenging research in developing countries can be partially overcome.

One way of compensating for these geographical limitations is to outsource technologies, know-how, and expertise in order to gain access to a diversified network of resources including—but not limited to—XR technologies, test subjects, and financial assets. The potential of distributing such resources across multiple countries (or continents), especially in developing nations, unfolds the possibility of overcoming socioeconomic barriers which, at times, prevent talented neurosurgeons and trainees from gaining access to useful tools for their own improvement, learning, and skill assessment. Additionally, disparities between different areas of the globe when it comes to education in neurosurgery can be addressed by developing XR-based applications that are readily accessible on the market, easy to use, and require low maintenance to operate [21,22].

Interestingly, we found that only eight studies (26%) employed HMDs as a type of holographic display, while 23 (74%) employed static monitors with a fixed point of view. While the latter enables a better control of environmental variables and user interaction as well as easing the development of XR systems without the need to register virtual on real imagery, the former allows participants more freedom of movement, a higher quality visual perception of the virtual environment (bigger field of view, three degrees of freedom head movements, higher pixel density, adjustment for vision limitations, etc.), and a more natural interaction with varying degrees of augmentation (i.e., the amount of virtual imagery superimposed on real imagery). Throughout the literature presented in this review, static monitors were found to be more commonly used in conjunction with haptic devices and more often associated with a more thorough UP assessment, with a greater number of metrics being involved than HMDs. Although less thoroughly investigated within this area, we argue that more studies presenting educational applications on commercial HMDs would supplement and strengthen the foundations of research presented in the existing literature involving devices such as the NeuroVR and the ImmersiveTouch. Moreover, because of their low cost, we believe that promoting the use of HMDs in particular could potentially enable departments all over the world—especially in developing countries—to carry out research on cranial neurosurgical practices by overcoming socioeconomic challenges related to the resources needed to purchase, maintain, and install more complex and advanced XR systems, such as NeuroVR. A quick comparison between advantages and disadvantages of the two technologies—fixed monitors and HMDs—is presented in Table 5.

A relevant challenge in designing user tests that yield meaningful impact in this specific field of research is that of participant recruitment; as with any other medical specialty field, it is important to have a sufficiently big dataset to analyze by involving a significant number of users, especially when carrying out between-subjects tests (e.g., residents vs. experienced neurosurgeons). Only seven of the 31 (23%) studies presented in this review collected data from 50 participants or more, and the overall average across all studies amounted to fewer than 31 participants; when only considering medical students, neurosurgical residents, and interns, who are the primary user base for such educational applications, the average drops to slightly above 24, with only five studies (16%) collecting data from 50 participants of these categories or more. This observation highlights the need for recruiting larger pools of test subjects. Again, developing XR applications that are scalable and accessible onto widespread commercial devices, such as HMDs, could help amplify both the cohort sizes and the overall number of studies, empowering the evidence base surrounding this topic.

In the present review, we arbitrarily divided neurosurgical education into assessment of skills, training or practicing, and acquisition of procedural knowledge. Studies considered here were then categorized according to their main subject of focus among these three aspects, albeit it is worth clarifying that not all papers address a single specific aspect of education and not all of them do so in the same way. An example is the possible approaches researchers can take in skill assessment: an XR system can be developed with the specific aim of enabling self-assessment and appreciation by the users themselves, or evaluation and selection by experienced neurosurgeons. The boundaries between these arbitrary labels can thus at times be blurry; nevertheless, we can still infer conclusions from the overall distribution of papers across different groups. Specifically, studies on procedural knowledge acquisition are the least common, focus on a variety of medical practices, and cover a total of six different devices (Table 2); this in turn suggests that more research addressing this particular aspect of education through longitudinal experiments is needed, with the aim of observing learning curves in test subjects when acquiring new skills.

Moreover, UX, despite being a component of major importance in the development of educational applications, has so far been focused on in a limited and non-scalable approach. As mentioned in previous sections, less than half of the papers presented here address the topic, and only one of them employs standardized, validated questionnaires to assess different measures of UX which, despite not being specifically related to the kind of procedure considered (ventriculostomy), allow for an easier interpretation and comparison of the proposed results with other—past and future—studies. Other publications present a varied and novel set of custom questionnaire items which delve to a certain extent into procedure-related aspects, or more generally assess a heterogeneous combination of UX factors, such as usefulness and realism. Although such experimental designs can yield meaningful results that can be used to estimate the impact of the study on the research field, a comparison with related work, as well as bias avoidance and formal validation of the chosen approach, can be challenging. This, in turn, may partially undermine the quality of the proposed conclusions, especially for those papers in which UX questionnaire items were proposed by the authors without any theoretical foundations to them. Of these types of survey items, task difficulty and consistency with real practices are particularly relevant when developing XR-based educational applications; however, only few studies (n = 4 in both cases) include them when evaluating the related proposed applications. Possible reasons for this are existing challenges in objectively defining task difficulty, and partial redundancies in perceived realism—which is at times addressed with more or less specificity, even multiple times in a single survey. In order to address this issue, standardized questionnaires (e.g., SUS, NASA TLX, and others) need to be consistently employed and, in future research, novel questionnaires that are more specific to the field of neurosurgery need to be developed.

Compared to UX, UP is the subject of most attention throughout the 31 papers presented in this review. User performance is a broad term that refers to relevant aspects of performed surgical simulations, such as eye–hand coordination, manual dexterity, success metrics, and kinematic coordination, to name a few. Most often, metrics of UP are assessed by experienced neurosurgeons or by test subjects themselves after the surgical simulation, which, despite any attempt at avoiding bias, can still be influenced by human factors and therefore lead to inaccuracies in assessment. Additionally, by involving human actors in the measurement and judgment of surgical performance, comparison of results across multiple studies (and even within longitudinal ones) may be biased, hence lowering the overall quality of the proposed conclusions.

A number of studies have dealt with this issue by employing metrics that are computed automatically—and oftentimes in real time—by the same system that the simulation is performed with; in particular, those focusing on more established types of procedure (i.e., tumor resection, ventriculostomy, aneurysm clipping) present a more structured and “advanced” set of metrics when compared to procedures considered in fewer studies. Possible explanations for this trend are the higher amount of research revolving around XR-based neurosurgical education within certain types of surgical practices, and also the lack of any type of optical tracking in the NeuroVR and ImmersiveTouch systems, which in turn partially enables an easier automatic assessment of UP. We can speculate that, in such cases as trigeminal rhizotomy, endoscopy, and cauterization, the adoption of detailed objective UP metrics that are assessed automatically is still underdeveloped and therefore future research could compensate for that. More in general, future research on XR applications to neurosurgical education can possibly focus on the replacement of humans with artificial intelligence (AI) systems in the measuring of UP. When comparing different subject groups, we show that not all UP metrics seem to distinguish experienced neurosurgeons from residents and medical students. Knowing which metrics to use in order to accurately assess skills can be a challenge, which is where machine learning comes into play. In future research, such an approach could aid in assessing performance by looking at overall performance instead of specific metrics. This has already been attempted by two studies included in this review [59,70], with the aim of accurately assigning users to their corresponding expertise level.

An essential contributing factor to the quality of the learning experience is the sense of realism as experienced by users when performing surgical simulations in an XR environment. Realism in this context refers to the fidelity of imagery, interaction, and context of an XR application, and quantifies how closely resembling a real-life scenario the visual (and tactile) stimuli are in the mind of the users. The more realistic the simulation is, the higher the quality of the education is [78]. This is currently addressed in present research through non-standardized questionnaires which, despite covering a broad range of facets within realism itself, have so far recorded inconclusive results on the matter. It is, in fact, unclear whether, when comparing subject groups with different levels of expertise (e.g., residents vs. experienced neurosurgeons vs. medical students), less experienced users find the simulation environment more realistic than experienced users, or vice versa. Significant impact may in this context be brought about with the use of surgical phantoms in simulated surgeries, a practice that has already been explored much in previous research—including that within the field of cranial neurosurgery—with solid conclusions stating their usefulness. By employing such phantoms in educational applications, thus implementing so-called augmented virtuality (AV), perceived realism of the experience can arguably benefit from the registration of the physical object with the virtual counterpart (also known as digital twin).

With the present systematic review, we aimed at shedding light on the plethora of recent research on the topic of extended reality applied to cranial neurosurgery. As shown in multiple studies [79,80,81], such technology can have a significant impact on the quality of education intended as training and practicing, skill assessment, and procedural knowledge acquisition. Not only does it enable immersive, realistic simulation of surgical practices in any place and at any time, but it also breaks the boundaries of traditional teaching by expanding it with detailed 3D models of the anatomy, haptic force feedback, automatic performance measurements, and more. While extensive research has already been carried out in other surgical specialties on the topic (including spinal neurosurgery), the specific field of cranial neurosurgery is still in its infancy when it comes to applying XR technologies to educational applications. Nevertheless, studies introduced here present solid results to prove the potential usefulness of such an approach, including when compared to or complementing traditional tutoring and teaching. In this perspective, less experienced users generally appreciate it more than the more experienced counterpart, with only a single study suggesting no difference between subject groups. Assessment of learning curves among trainees such as neurosurgical residents and medical students, as well as comparisons with that of experienced surgeons, show that in surgeries with a lesser degree of variability in their operative conditions (such as ventriculostomy), the performance improvement over time tends to be greater than that in other surgeries (in particular, tumor resection).

Ventriculostomy is typically uniform in its course; thus, successful outcomes resulting from this procedure are highly relying on anatomical knowledge and experience, unlike tumor resection surgeries where monotony is practically absent, which ascribes the skills and dexterity of the surgeon a much bigger role. Hence, improvements are hard to visualize on the modest follow-up timeline provided by most longitudinal studies. In fact, it was found that UP improvements in tumor resection were either non-significant or much more subtle [58,59] as compared to those witnessed for ventriculostomy [19,65]. Along the same lines, in both tumor resection studies [58,59], more training sessions (four and five, respectively) were required to detect even the small improvements in UP, as compared to studies reporting on ventriculostomy procedures, where a single training session was sufficient to significantly improve UP both immediately [19,65] and at a 47-day average follow-up [19]. In summary, the findings described seem to indicate that some surgical procedures may require longer training durations as compared to others, and that studies considering such procedures ought to expand their timeline in order for the desired outcomes to be correctly appreciated.

In our opinion, the true value of this innovative technology ultimately lies in improvement of both quality of patient care and operative outcomes. Despite the fact that only a small number of studies (n = 3) projected the benefits of training in XR settings on live-patient operator performances [19,62,69], the results obtained thus far all proved promising, with increased overall success rates in favor of XR-trained residents. This provides insightful knowledge on the effectiveness of XR simulation in improvement of patient outcomes, and highlights the need for taking the research on this topic one step further with a more patient-centered focus and a larger pool of both participants and patients.

### 5.1. Limitations

As for the limitations to this review, an exclusively user-centered approach was adopted to study the impact of this technology on the field of interest, while system-related performance metrics, constrained to the capabilities of the technologies employed, were not considered. Additionally, we only performed a narrative synthesis of the major trends in current research, while meta-analysis of the data was not attempted due to the high heterogeneity with regards to populations, comparators, and outcome metrics.

### 5.2. Future Perspective

Finally, we suggest that research employing diversified XR technologies (such as HMDs) to monitor mid- to long-term improvements in trainee surgical simulation performance through longitudinal within-subjects studies could help confirm conclusions presented in previous work, both in the field of cranial neurosurgery and in the broader field of education in medicine. Moreover, we propose that an accurate analysis of biomechanical factors, measured automatically by capturing body and hand movements in real time during surgical simulation, could improve the evaluation of users’ dexterity and eye–hand coordination when performing specific surgical procedures. Through a comparison of trainee skills with those of experienced neurosurgeons, appreciation of learning curves in an extended timespan would be enabled and, ultimately, access to high-level education would be open to residents and medical students all over the world. Eventually, detailed and precise computation of performance metrics via the implementation of AI in XR systems would decrease the need for expert assessment when monitoring trainee improvements, thus facilitating the acquisition of procedural knowledge related to cranial neurosurgical procedures.

## Figures and Tables

**Table 1:** Eligibility criteria. Summary of criteria for inclusion of studies in the present review, based on relevant attributes and the PICO (population, intervention, comparators, outcome) model.

Criteria	Inclusion	Exclusion
Study type	Empirical studies presenting quantitative data	Narrative or non-empirical studies (reviews, editorials, opinions)
Year of publishing	2013–2022	Before 2013
Language	English	All other languages
Population	n/a	n/a
Device	Stereoscopic, off-the-shelf displays	Mobile-based
Intervention	Procedural skill acquisition in cranial neurosurgery	Spinal neurosurgery, other medical specialties and other application domains (e.g., patient education, surgical navigation, preoperative planning)
Comparator	n/a	n/a
Outcome	Performance metrics and/or user experience	n/a

**Table 2:** Overview of the 31 included studies. Baseline characteristics and data extracted from the studies.

ID	Country	Population	Domain	Procedure	Device	XR Type	Haptics	RoB
Alaraj 2015 [46]	USA	17 R	Practice	Aneurysm clipping	ImmersiveTouch †	VR	YES	1
Alotaibi 2015 [47]	Canada	6 JR, 6 SR, 6 E	Skill assessment	Tumor resection	NeuroVR †	VR	YES	3
AlZhrani 2015 [48]	Canada	9 JR, 7 SR, 17 E	Skill assessment	Tumor resection	NeuroVR †	VR	YES	4
Ansaripour 2019 [49]	UK	6 MS, 12 R, 4 E	Practice	Microsurgical tasks	NeuroVR †	VR	N/A	4
Azarnoush 2015 [50]	Canada	1 JR, 1 E	Skill assessment	Tumor resection	NeuroVR †	VR	YES	3
Azimi 2018 [51]	USA	10 NP	Learning	Ventriculostomy	HoloLens ‡	AR	NO	2
Breimer 2017 [52]	Canada	23 R, 3 F	Practice	ETV	NeuroVR †	VR	YES	2
Bugdadi 2018 [53]	Canada	10 SR, 8 JR	Skill assessment	Tumor resection	NeuroVR †	VR	YES	3
Bugdadi 2019 [54]	Canada	6 E	Practice	Subpial tumor resection	NeuroVR †	VR	YES	2
Cutolo 2017 [55]	Italy	3 E	Practice	Surgical access, tumor detection	Sony HMZ-T2 ‡	AR	NO	2
Gasco 2013 [56]	USA	40 MS, 13 R	Learning	Bipolar hemostasis	ImmersiveTouch †	VR	YES	2
Gelinas-Phaneuf 2014 [57]	Canada	10 MS, 18 JR, 44 SR	Skill assessment	Meningioma resection	NeuroVR †	VR	YES	5
Holloway 2015 [58]	USA	71 MS, 6 JR, 6 SR	Learning	GBM resection	NeuroVR †	VR	YES	3
Ledwos 2022 [59]	Canada	12 MS, 10 JR, 10 SR, 4 F, 13 E	Practice	Subpial tumor resection	NeuroVR †	VR	YES	4
Lin 2021 [60]	China	30 I	Learning	Lateral ventricle puncture	HTC VIVE Pro ‡	VR	YES	5
Patel 2014 [61]	USA	20 MS	Learning	Detection of objects in brain cavity	ImmersiveTouch †	VR	YES	5
Perin 2021 [62]	Italy	2 JR, 1 F, 4 E	Practice	Aneurysm clipping	Surgical Theater ‡	VR	YES	4
Roh 2021 [63]	South Korea	31 R	Learning	Cranial neurosurgical procedures of unspecified type	Oculus Quest 2 ‡	AV	NO	2
Roitberg 2015 [30]	USA	64 MS, 10 MS, 4 JR	Skill assessment	Cauterization and detection of objects in brain cavity	ImmersiveTouch †	VR	YES	3
Ros 2020 [64]	France	1 st exp. 176 MS, 2nd exp. 80 MS	Learning	EVD placement	Samsung Gear VR ‡	VR	NO	5
Sawaya 2018 [27]	Canada	14 R, 6 E	Skill assessment	Tumor resection	NeuroVR †	VR	YES	3
Sawaya 2019 [28]	Canada	6 MS, 6 JR, 6 SR, 6 E	Skill assessment	Tumor resection	NeuroVR †	VR	YES	4
Schirmer 2013 [65]	USA	10 R	Learning	Ventriculostomy	ImmersiveTouch †	VR	YES	4
Shakur 2015 [66]	USA	44 JR, 27 SR	Skill assessment	Trigeminal Rhizotomy	ImmersiveTouch †	VR	YES	3
Si 2019 [67]	China	10 NP	Learning	Tumor resection	HoloLens ‡	AR	YES	2
Teodoro-Vite 2021 [68]	Mexico	6 R, 6 E	Practice	Aneurysm clipping	Unspecified ‡	VR	YES	3
Thawani 2016 [69]	USA	6 JR	Practice	Endoscopic surgery	NeuroVR †	VR	YES	5
Winkler-Schwartz 2016 [31]	Canada	16 MS	Skill assessment	Tumor resection	NeuroVR †	VR	YES	3
Winkler-Schwartz 2019 [70]	Canada	12 MS, 10 JR, 10 SR, 4 F, 14 E	Skill assessment	Subpial tumor resection	NeuroVR †	VR	YES	2
Winkler-Schwartz 2019 [71]	Canada	16 MS	Skill assessment	Tumor resection	NeuroVR †	VR	YES	4
Yudkowsky 2013 [19]	USA	11 JR, 5 SR	Practice	Ventriculostomy	ImmersiveTouch †	AV	YES	3

RoB = risk of bias; R = residents; JR = junior residents; SR = senior residents; E = experts; MS = medical students; NP = naïve participants; F = fellows; I = interns; † = relying on flat, static monitors; ‡ = relying on head-mounted displays.

**Table 3:** User performance. List of studies addressing quantitative metrics of UP, along with related relevant experimental design features.

Study ID	Outcome	vs. No-XR	Longitudinal	Training Level Comparison
Alotaibi 2015	UP and UX	NO	NO	YES
AlZhrani 2015	UP	NO	NO	YES
Ansaripour 2019	UP	NO	YES	NO
Azarnoush 2015	UP	NO	NO	YES
Azimi 2018	UP and UX	NO	YES	YES
Bugdadi 2018	UP	NO	NO	YES
Bugdadi 2019	UP and UX	NO	NO	NO
Cutolo 2017	UP	NO	YES	NO
Gelinas-Phaneuf 2014	UP and UX	NO	NO	YES
Holloway 2015 *	UP	YES	NO	YES
Ledwos 2022 *	UP	YES	NO	YES
Lin 2021	UP and UX	NO	YES	NO
Patel 2014	UP	NO	YES	NO
Perin 2021	UP and UX	NO	YES	NO
Roitberg 2015	UP	NO	NO	YES
Ros 2020	UP	YES	YES	NO
Sawaya 2018	UP	NO	NO	YES
Sawaya 2019	UP	NO	NO	YES
Schirmer 2013 *	UP	YES	NO	YES
Shakur 2015	UP	NO	NO	YES
Teodoro-Vite 2021	UP and UX	NO	NO	YES
Thawani 2016	UP	YES	YES	NO
Winkler-Schwartz 2016	UP	NO	NO	YES
Winkler-Schwartz 2019	UP	NO	NO	NO
Winkler-Schwartz 2019	UP	NO	NO	NO
Yudkowsky 2013 *	UP and UX	YES	NO	YES

* Studies that also assessed longitudinal difference in improvement between subject groups (i.e., different training levels).

**Table 4:** User experience. List of studies addressing quantitative metrics of UX, along with related relevant experimental design features.

Study ID	Outcome	Usefulness	Realism	Questionnaire items
Alaraj 2015	UX	YES	YES	Binary questions + Likert scales
Alotaibi 2015	UP and UX	YES	YES	Likert scales
Azimi 2018	UP and UX	NO	NO	Likert scales
Breimer 2017	UX	NO	YES	Likert scales + open comments
Bugdadi 2019	UP and UX	NO	NO	Likert scales
Gasco 2013	UX	YES	NO	Binary questions + Likert scales
Gelinas-Phaneuf 2014	UP and UX	YES	YES	Likert scales + open comments
Lin 2021	UP and UX	YES	NO	Binary questions + Likert scales + open comments
Perin 2021	UP and UX	YES	YES	Binary questions + Likert scales + open comments
Roh 2021	UX	YES	YES	Likert scales + open comments
Si 2019	UX	YES	YES	Likert scales
Teodoro-Vite 2021	UP and UX	NO	YES	Likert scales
Yudkowsky 2013	UP and UX	YES	YES	Likert scales + open comments

**Table 5:** A few of the advantages and disadvantages of static, flat monitors vs. head-mounted displays, in the context of the present review.

Technology	Advantages	Disadvantages
Fixed monitors	Better precision	Expensive
	Easier registration	Limited motion range
	More control over experiments	Not immersive
HMDs	Relatively affordable	Poor research coverage
	Enables AR	Calibration required
	3 degrees of freedom	

## Data Availability

All data used were openly available.

## Supplementary Materials

The following supporting information can be downloaded at https://www.mdpi.com/article/10.3390/s22166067/s1, File S1: PRISMA 2020 checklist, title, File S2: elaborate description of search strategy creation, File S3: Search strategy and queries used in each database, File S4: Extended Risk of Bias table.

## Abbreviations

AI	Artificial intelligence
AR	Augmented reality
AV	Augmented virtuality
ETV	Endoscopic third ventriculostomy
HMD	Head-mounted display
MR	Mixed reality
NOS-E	Newcastle–Ottawa Scale-Education
OR	Operating room
PICO	Population, intervention, comparators, outcome
PRISMA	Preferred Reporting Items for Systematic Reviews and Meta-Analyses
PROSPERO	International Prospective Register of Systematic Reviews
SUS	System Usability Scale
TLX	Task load index
UP	User performance
UX	User experience
VR	Virtual reality
XR	Extended reality
