# Supplementary material for: Extended Reality in Neurosurgical Education: A Systematic Review
Source: Sensors (Basel). 2022 Aug 14;22(16):6067. doi: 10.3390/s22166067 (PMC9414210; doi:10.3390/s22166067)
Supplement: Supplementary file 1 [file sensors-22-06067-s001.zip › Supplementary_Files/Supplementary_file_3_queries_in_each_database.pdf]

Databases	Descriptors
Pubmed	("virtual reality"[Title/Abstract] OR VR[Title/Abstract] OR AR[Title/Abstract] OR "augmented reality"[Title/Abstract] OR "mixed reality"[Title/Abstract] OR MR[Title/Abstract] OR "extended reality"[Title/Abstract] OR XR[Title/Abstract]) AND (neurosurgery[Title/Abstract] OR neurosurgical[Title/Abstract] OR "cranial surgery"[Title/Abstract] OR "brain surgery"[Title/Abstract]) AND (education[Title/Abstract] OR training[Title/Abstract] OR practice[Title/Abstract] OR learning[Title/Abstract])
IEEE Xplore	(("Abstract":"virtual reality" OR "Abstract":VR OR "Abstract":"augmented reality" OR "Abstract":AR OR "Abstract":"mixed reality" OR "Abstract":MR OR "Abstract":"extended reality" OR "Abstract":XR) AND ("Abstract":neurosurgery OR "Abstract":neurosurgical OR "Abstract":"brain surgery" OR "Abstract":"cranial surgery") AND ("Abstract":education OR "Abstract":training OR "Abstract":practice OR "Abstract":learning))<br><br>(("Document Title":"virtual reality" OR "Document Title":VR OR "Document Title":"augmented reality" OR "Document Title":AR OR "Document Title":"mixed reality" OR "Document Title":MR "Document Title":"extended reality" OR "Document Title":XR) AND ("Document Title":neurosurgery OR "Document Title":neurosurgical OR "Document Title":"brain surgery" OR "Document Title":"cranial surgery") AND ("Document Title":education OR "Document Title":training OR "Document Title":practice OR "Document Title":learning))
ACM DL	(Abstract:("virtual reality") OR Abstract:(VR) OR Abstract:("augmented reality") OR Abstract:(AR) OR Abstract:("mixed reality") OR Abstract:(MR) OR Abstract:("extended reality") OR Abstract:(XR)) AND (Abstract:(neurosurgery) OR Abstract:(neurosurgical) OR Abstract:("brain surgery") OR Abstract:("cranial surgery")) AND (Abstract:(education) OR Abstract:(training) OR Abstract:(practice) OR Abstract:(learning))<br><br>(Title:("virtual reality") OR Title:(VR) OR Title:("augmented reality") OR Title:(AR) OR Title:("mixed reality") OR Title:(MR) OR Title:("extended reality") OR Title:(XR)) AND (Title:(neurosurgery) OR Title:(neurosurgical) OR Title:("brain surgery") OR Title:("cranial surgery")) AND (Title:(education) OR Title:(training) OR Title:(practice) OR Title:(learning))
Web of Science	(AB=("virtual reality") OR AB=(VR) OR AB=("augmented reality") OR AB=(AR) OR AB=("mixed reality") OR AB=(MR) OR AB=("extended reality") OR AB=(XR)) AND (AB=(neurosurgery) OR AB=(neurosurgical) OR AB=("brain surgery") OR AB=("cranial surgery")) AND (AB=(education) OR AB=(practice) OR AB=(training) OR AB=(learning))<br><br>(TI=("virtual reality") OR TI=(VR) OR TI=("augmented reality") OR TI=(AR) OR TI=("mixed reality") OR TI=(MR) OR TI=("extended reality") OR TI=(XR)) AND (TI=(neurosurgery) OR TI=(neurosurgical) OR TI=("brain surgery") OR TI=("cranial surgery")) AND (TI=(education) OR TI=(practice) OR TI=(training) OR TI=(learning))
Scopus	(TITLE-ABS ("virtual reality" OR vr OR "augmented reality" OR ar OR "mixed reality" OR mr OR "extended reality" OR xr) AND TITLE-ABS (neurosurgery OR neurosurgical OR "brain surgery" OR "cranial surgery") AND TITLE-ABS (practice OR education OR training OR learning))
