# Supplementary material for: Extended Reality in Neurosurgical Education: A Systematic Review
Source: Sensors (Basel). 2022 Aug 14;22(16):6067. doi: 10.3390/s22166067 (PMC9414210; doi:10.3390/s22166067)

Article code	Representativeness of intervention group (1p)	Selection of comparison group (1p)	Comparability of comparison group (2p)	Study retention (1p)	Blinding of assessment (1p)	Overall scores (6p)
Alaraj 2015	0	0	0	0	1	1
Alotaibi 2015	0	0	1	1	1	3
AlZhrani 2015	0	0	2	1	1	4
Ansaripour 2019	1	1	1	0	1	4
Azarnoush 2015	0	0	1	1	1	3
Azimi 2018	0	0	0	1	1	2
Breimer 2017	0	0	0	1	1	2
Bugdadi 2018	0	0	1	1	1	3
Bugdadi 2019	0	0	0	1	1	2
Cutolo 2017	0	0	0	1	1	2
Gasco 2013	0	0	0	1	1	2
Gelinas-Phaneuf 2014	1	0	2	1	1	5
Holloway 2015	0	0	1	1	1	3
Ledwos 2022	1	0	1	1	1	4
Lin 2021	1	1	1	1	1	5
Patel 2014	0	1	2	1	1	5
Perin 2021	0	1	1	1	1	4
Roh 2021	1	0	0	0	1	2
Roitberg 2015	1	0	0	1	1	3
Ros 2020	1	1	2	0	1	5
Sawaya 2018	0	0	1	1	1	3
Sawaya 2019	1	0	1	1	1	4
Schirmer 2013	1	0	1	1	1	4
Shakur 2015	1	0	0	1	1	3
Si 2019	0	0	0	1	1	2
Teodoro-Vite 2021	0	0	1	1	1	3
Thawani 2016	1	1	1	1	1	5
Winkler-Schwartz 2016	1	0	0	1	1	3

Winkler-Schwartz 2019	1	0	0	0	1	2
Winkler-Schwartz 2019	1	0	1	1	1	4
Yudkowsky 2013	1	0	0	1	1	3

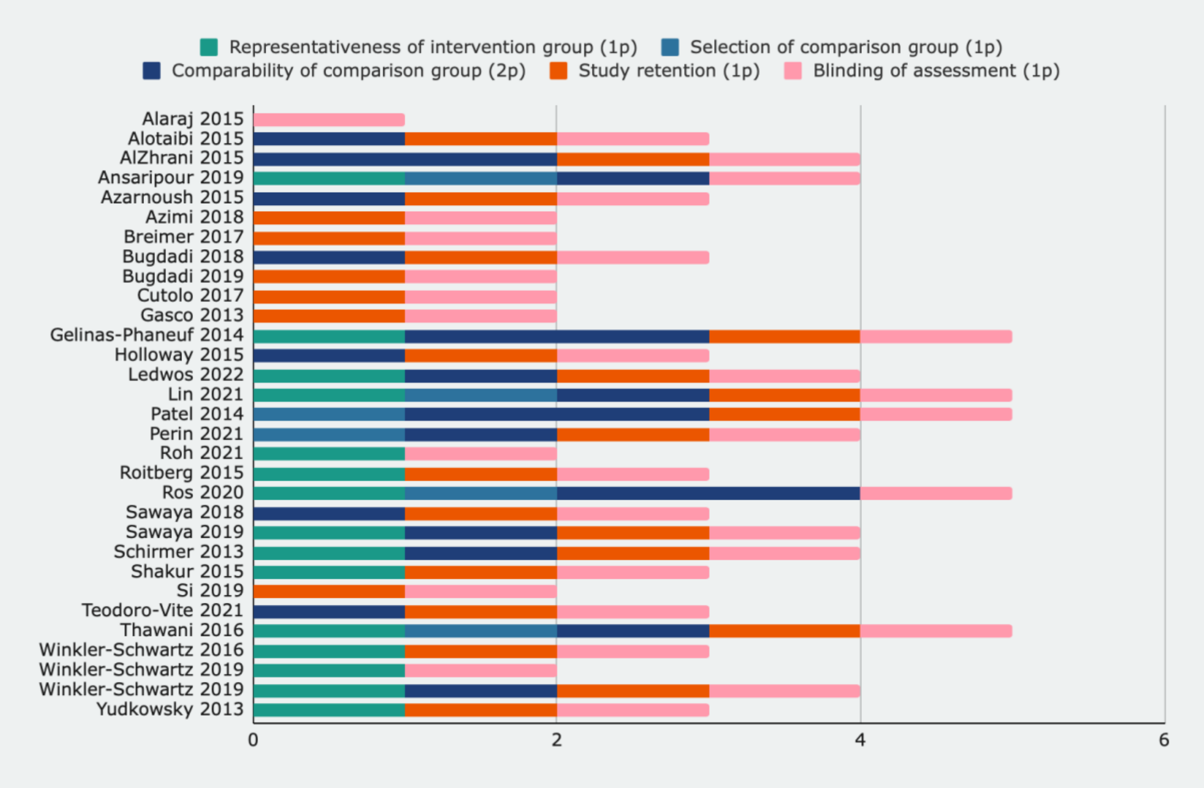

Supplement: Supplementary file 1 [file sensors-22-06067-s001.zip › Supplementary_Files/Supplementary_file_4_Extended_Risk_of_Bias_table.pdf]
