# Supplementary material for: Extended Reality in Neurosurgical Education: A Systematic Review
Source: Sensors (Basel). 2022 Aug 14;22(16):6067. doi: 10.3390/s22166067 (PMC9414210; doi:10.3390/s22166067)
Supplement: Supplementary file 1 [file sensors-22-06067-s001.zip › Supplementary_Files/Supplementary_file_2_elaborate_description_of_search_strategy_creation.pdf]

Elaborate description of the development of the search strategy used in our systematic review.

In information retrieval, precision is a measure of true positive rate. Recall is a measure of true negative rate. Of the papers retrieved, precision is the fraction of them that are relevant targets of the search. Irrelevant papers returned by the search are false positives and they lower precision. Of the relevant papers that exist in the database, recall is the fraction of them that are retrieved during the search. False negatives, that is papers that are relevant but missed in the search, lower recall rate.

With this in mind, database interrogation started with a broad and unpolished set of keywords and fields, according to our research question and intended domain of application. Terms such as XR and all its variants, (cranial) neurosurgery, education and related synonyms were therefore initially considered for a broad exploration of the existing literature. This was performed mainly on titles and abstracts, but also included keywords and full-text searches. In particular, several different queries were built and tested on multiple engines; the corresponding number of resulting papers was then recorded for a later comparison with other queries, until a total amount that encompassed a narrow enough collection was reached. At this stage, several sources were excluded when no export function was available, when paywalls were present and when databases were interrogated redundantly.

To increase the recall of our methodology, the structure of the query was subsequently more formally defined in detail. Three parts in particular were subject of focus in this context: what technology was employed, the specific domain of application of the research, and the purpose of the studies presented (their primary use case). Concerning the technology, we selected eight keywords and acronyms that are relevant to our research questions, i.e. AR, VR, MR, XR and their extended forms. As for the application, upon reviewing our previous test searches and considering our domain of interest, we agreed on a set of four keywords that includes ``neurosurgery'', ``neurosurgical'', ``brain surgery'' and ``cranial surgery''. Finally, in order to select meaningful keywords for the purpose of the studies, initially several possible options were listed for each of three arbitrary categories - namely training, planning and executing - along with their variants and synonyms. Each option was then ``tested'' in a specific database interrogation by keeping the first two parts (technology and application) of the query constant and changing the value of a single keyword in its last part, and recording the resulting number of papers retrieved. The four most ``popular'' options belonging to the training category were selected for the final query, and these were ``training'', ``education'', ``practice'', ``learning''. All 16 keywords to be included in the query were searched for in both titles and abstracts by using specific command words provided by the search engines; when no option for interrogating both title and abstract at the same time was present, multiple interrogations were made on the engine by switching the two corresponding command words, and results were integrated with the logical operator “OR”.

To increase the precision of our methodology, among all different search engines considered in our initial exploration five of them were selected for the reviews as they cover

both the fields of medicine and engineering, and they interrogate complementary (at least partially) databases. This ensured that potentially valuable literature was not unnecessarily excluded from the search.
